# The taxonomy of two uncultivated fungal mammalian pathogens is revealed through phylogeny and population genetic analyses

**DOI:** 10.1038/s41598-021-97429-7

**Published:** 2021-09-13

**Authors:** Raquel Vilela, Marianne Huebner, Camila Vilela, Gabriella Vilela, Bruno Pettersen, Claudia Oliveira, Leonel Mendoza

**Affiliations:** 1 Biomedical Laboratory Diagnostics, Michigan State University, East Lansing, MI 48824 USA; 2 Faculty of Pharmacy, Federal University of Minas Gerais, Belo Horizonte, 31270 Brazil; 3 Department of Statistics and Probability, Michigan State University, East Lansing, MI 48824 USA; 4 Microbiology and Molecular Genetics, Michigan State University, East Lansing, MI 48824 USA

**Keywords:** Biological techniques, Ecology, Evolution, Genetics, Microbiology, Molecular biology, Diseases, Medical research

## Abstract

Ever since the uncultivated South American fungal pathogen *Lacazia loboi* was first described 90 years ago, its etiology and evolutionary traits have been at the center of endless controversies. This pathogen infects the skin of humans and as long believed, dolphin skin. However, recent DNA analyses of infected dolphins placed its DNA sequences within *Paracoccidioides* species. This came as a surprise and suggested the human and dolphin pathogens may be different species. In this study, population genetic analyses of DNA from four infected dolphins grouped this pathogen in a monophyletic cluster sister to *P. americana* and to the other *Paracoccidioides* species. Based on the results we have emended the taxonomy of the dolphin pathogen as *Paracoccidioides cetii* and *P. loboi* the one infecting human. Our data warn that phylogenetic analysis of available taxa without the inclusion of unusual members may provide incomplete information for the accurate classification of anomalous species.

## Introduction

In 1931 Jorge Lôbo^1^ in Recife, Brazil, reported an unusual yeast-like fungal pathogen in an Amazonian patient with lumbo-sacral skin lesions. Later other studies found this pathogen resisted culture^2–4^. In 1975, Migaky et al.^5^, reported a similar skin disease in dolphins dwelling the USA coasts, and based on its phenotypic features in the infected tissues and its uncultivated nature, they believed to be the same etiologic agent as that described in Brazilian humans^2–5^. Thereafter, the disease in humans was found restricted to Latin American countries, from Mexico to Argentina^2^, and in dolphins inhabiting the coastal areas of the Americas and Japan^5–7^. Due to its uncultivated nature, placement of this unusual pathogen within a particular taxon was always contentious^2,3,7,8^. Therefore, the pathogen as well as the disease were known under a long list of binomials and disease names including Jorge lobos’ disease, lobomycosis and more recently lacaziosis^2,3,9^. Based on phenotypic features in the infected tissues, early investigators believed the pathogen was related to the genus *Paracoccidioides* and thus, at one point, it was named *P. loboi*^10,11^. However, the proposal was challenged by many due to the pathogens’ intractability to culture and to the fact that it is restricted to the subcutaneous tissues, both features contrasting to that in *Paracoccidioides* species^2,8^. After a long list of unsuccessful names^2,9–12^, based on taxonomic nomenclatural issues, the binomial *Lacazia loboi* was introduced^8^.

The first phylogenetic study of genomic DNA extracted from a Brazilian patient with skin disease, clustered this uncultivated pathogen of humans with the dimorphic Onygenales, closely related to *P. brasiliensis*^13^. This was an expected outcome, since its phenotypic features in the skin of infected patients somehow, resembles the phenotype observed in cases of systemic paracoccidioidomycosis^14,15^. Further phylogenetic analysis using several rDNA and partial coding DNA sequences, recovered from numerous human cases in Brazil, supported the finding^16,17^. The latter studies showed statistical support for the genus *Lacazia* to be the etiologic agent of the subcutaneous disease in humans and dolphins. This notion was first challenged by Rotstein et al.^18^, with genomic DNA extracted from a USA bottlenose dolphin (*Tursiops truncatus*) with skin disease. The 28S rDNA sequence, amplified from the yeast-like cells in the infected dolphin showed higher identity with *P. brasiliensis*. Almost concomitantly, five groups in Brazil^19^, Japan^20,21^, Spain^22^, and the USA^23^ using DNA extracted from several species of dolphins with “lacaziosis” (*Lagenorhynchus obliquidens*, *T. aduncus*, and *T. truncatus*) confirmed the finding. These studies placed the DNA sequences from infected dolphins within *Paracoccidioides* species and away from that of *L. loboi* in humans. Based on these reports, it was predicted that the etiologic agent of the uncultivated skin pathogen of dolphins was probably a new *Paracoccidioides* species^20–23^. Moreover, because the uncultivated human pathogen shared common phenotypic features with that of dolphins, it was suggested the human pathogen possibly a *Paracoccidioides* species as well^12,23^. Therefore, Vilela and Mendoza^12^ introduced *P. brasiliensis* var*. ceti* as a new variety in the genus, to differentiate the dolphin pathogen from the one causing skin disease in humans.

To investigate the patterns of genetic diversity between the uncultivated skin pathogen of dolphin, *Paracoccidioides* species and *L. loboi*, we conducted phenotypic, phylogenetic and population genetic analyses using rDNA (ITS) and partial DNA coding sequences extracted from four dolphins swimming USA coastal areas with the disease and homologous DNA sequences in the data base. In these analyses the pathogen of dolphin grouped in a monophyletic cluster sister to *P. americana* and in turn, both species were sister to *P. brasiliensis*, *P. restrepiensis* and *P. venezuelensis*; whereas, the human pathogen formed a monophyletic cluster sister to *P. lutzii*. Based on this finding we have emended the taxonomy of both pathogens, now known as *P. cetii* and *P. loboi*.

## Results

### Phenotypic traits

**Figure 1:**
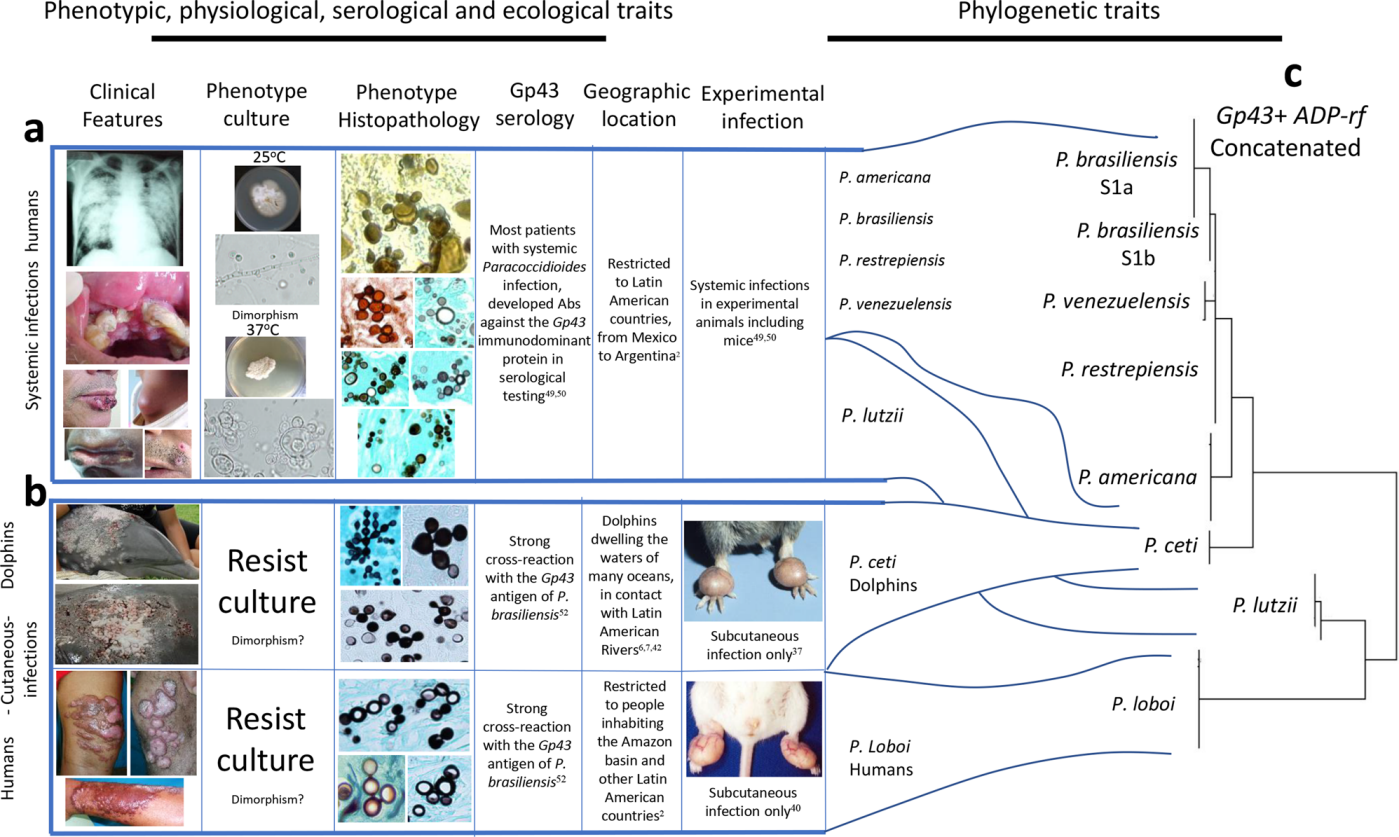
Clinical features, phenotypic, physiological, ecological, and other traits, group *Paracoccidioides* species into two clusters (**a**,**b**). Panel (**c**) display the phylogenetic analysis of 59 *Gp43* and *ADP-rf Paracoccidioides* species concatenated partial DNA sequences (including four USA dolphin). The phenotypic features shared by *P. americana*, *P. brasiliensis* (sensu stricto), *P. lutzii*, *P. restrepiensis* and *P. venezuelensis* is shown in panel “a”. Panel “b” comprises common phenotypic features shared by *P. cetii* and *P. loboi*. The phylogenetic traits (Panel **c**) using *Gp43* and *ADP-rf* concatenated data in phylogenetic analysis, placed members of both clusters (**a**,**b**) on inverted phylogenetic groups. For instance, *P. lutzii* grouped with *P. loboi* whereas *P. cetii* clustered with the other *Paracoccidioides* species. However, without the inclusion of *P. cetii* DNA sequences, *P. loboi* would appear as an independent genus, as described by others^15,16,24,25^. (Clinical picture in panel **b** were courtesy of Drs. J. St. Leger, G. D. Bossart, L. Ajello, and P. Rosa).

The phenotypic traits of *Paracoccidioides* species in this study grouped these pathogens into two clusters (Table 1). Except for a common *Gp43* antigen, recognized by the anti-*Gp43*-IgG antibodies (brown cell) present in infected hosts species (Table 1), the remaining 19 phenotypic traits showed *P. americana*, *P. brasiliensis*, *P. lutzii*, *P. restrepiensis* and *P. venezuelensis* sharing identical phenotypic traits (blue cells). *Paracoccidioides cetii* and *P. loboi* displayed contrasting phenotypic features with the above species (green and yellow cells), but similar traits with some differences between them (yellow cells). For instance, *P. cetii* is found causing disease in dolphins in many oceans (yellow cell) and, although *P. loboi* shares some phenotypes with *Paracoccidioides* species (blue cells), it displays numerous features in common with *P. cetii* (green cells). Figure 1 was assembled utilizing clinical and laboratorial data collected in our facilities. The phenotypic traits included their cultivated or uncultivated nature, morphological features in the infected tissues, and the capacity of causing systemic or subcutaneous infections. These phenotypic traits grouped the seven species in this study into two clusters (Fig. 1a,b). One cluster contains all known *Paracoccidioides* species causing systemic paracoccidioidomycosis in humans (Fig. 1a), whereas the other cluster comprised *P. cetii* and *P. loboi* causing subcutaneous infection in dolphins and humans (Fig. 1b). Figure 1c shows that loss of the ability to grow in culture is a paraphyletic trait in phylogenetic analysis among *Paracoccidioides* species.

**Table 1:**
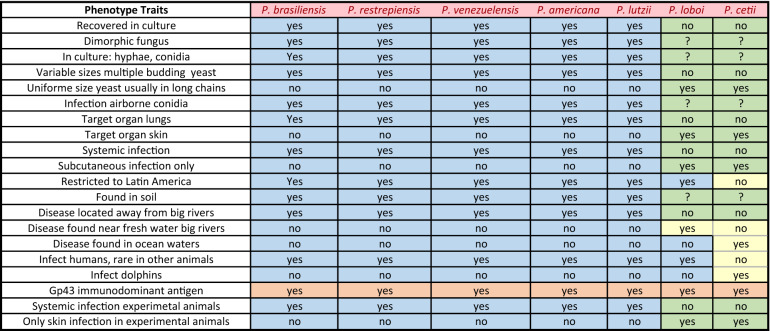
Phenotypic traist of *Paracoccidioides* species.

### Principal component analysis (PCA)

**Figure 2:**
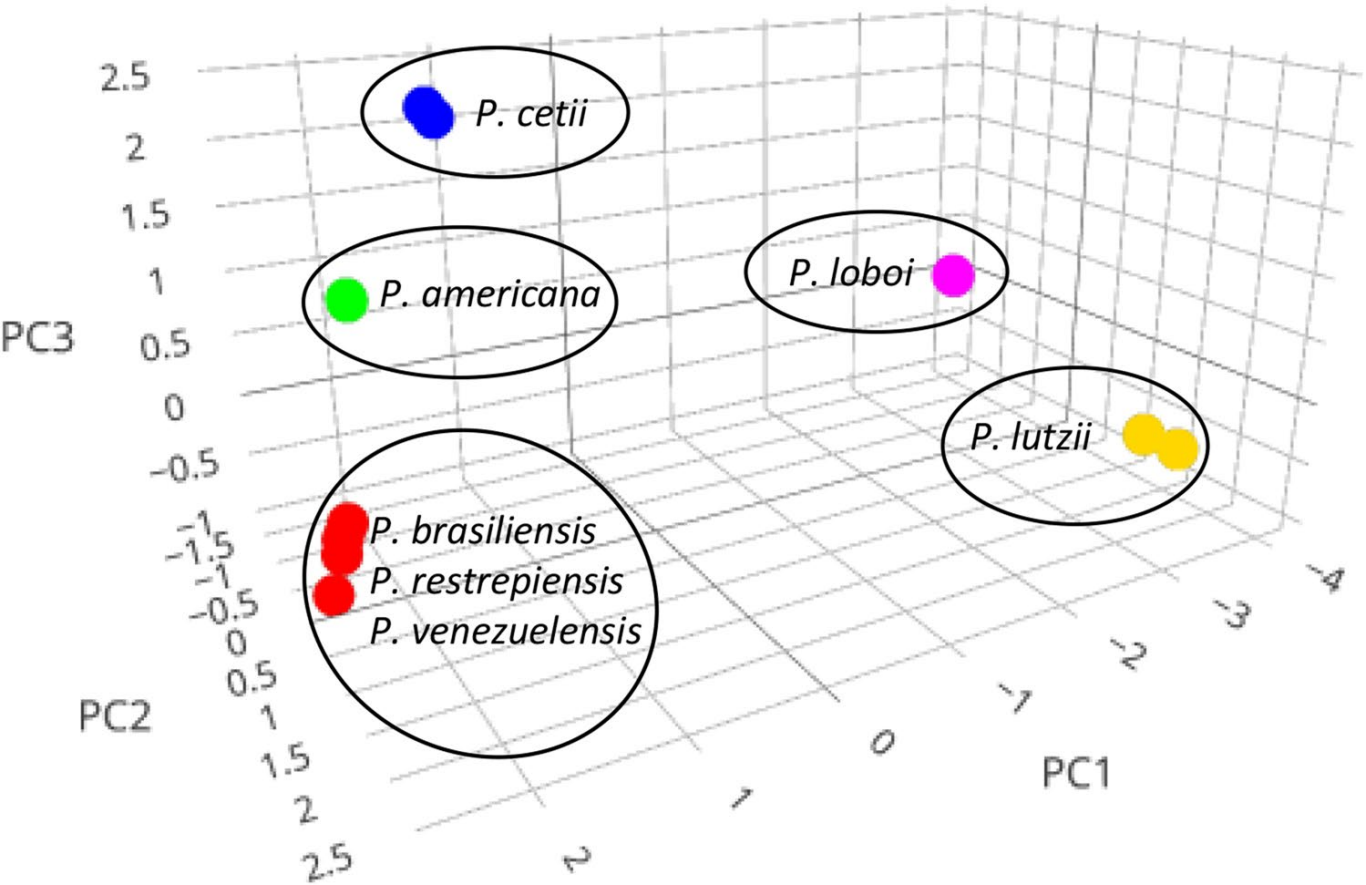
Graphical output of the *Gp43* DNA sequences PCA cluster analysis (total variation 90.3%, or 83.6% for PC1 (71.0%) and PC2 (12.6%)). The scatterplot shows the PC1, PC2 and PC3 results grouping *Paracoccidioides* species in five independent clusters including *P. americana* (green), *P. cetii* (blue), *P. loboi* (pink), and *P. lutzii* (yellow). *Paracoccidioides brasiliensis* (sensu stricto), *P. restrepiensis*, and *P. venezuelensis* appeared together in one of the clusters (red).

The *Gp43* partial DNA sequences (*P. americana*, n = 9; *P. brasiliensis*, n = 15; *P. cetii* n = 5; *P. loboi* n = 16; *P. lutzii*, n = 10; *P. restrepiensis* n = 10; *P. venezuelensis*, n = 5) (Fig. 2) (Table S1) using three components (PC1, PC2 and PC3) grouped *Paracoccidioides* species into five populations corresponding to some of the above species (Fig. 2). The three principal components accounted for 71.0%, 12.6%, and 6.7%, respectively, with cumulative variation explaining 90.3% of the interrogated DNA sequences, indicating the relationship of individuals present in each cluster is reliable (Fig. 2). *Paracoccidioides loboi* (pink) and *P. lutzii* (yellow) were discriminated by PCA, placing them in independent clusters on the lower right section of Fig. 2. *Paracoccidioides americana* (green) and *P. cetii* (blue) DNA sequences were also discriminated into two clusters on the upper left section of the graphic (Fig. 2). The remaining species including *P. brasiliensis*, *P. restrepiensis* and *P. venezuelensis* (red) formed a single cluster located at the lower left section of the graphic.

### Haplotype analysis

**Figure 3:**
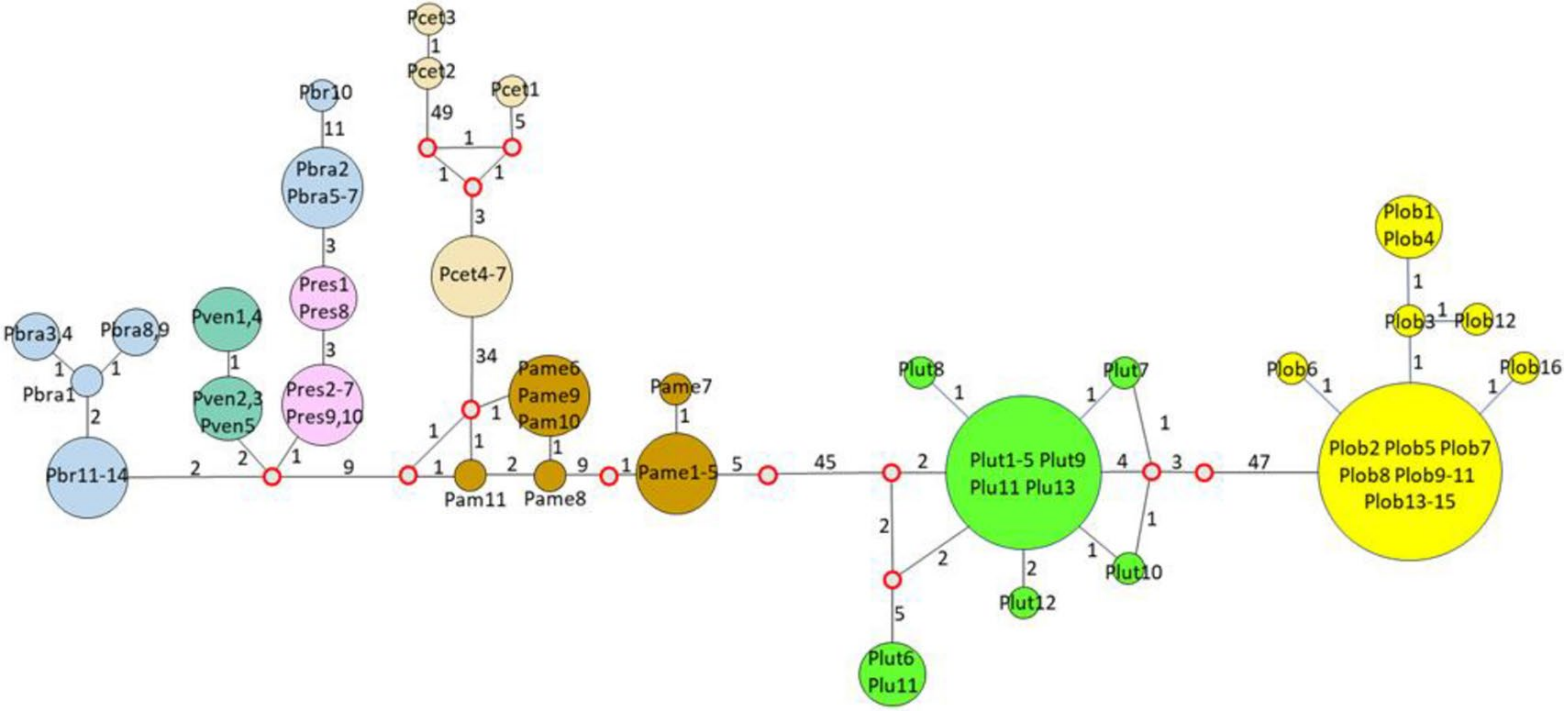
Neighbor-joining analysis of the *Gp43* partial DNA sequences revealed several haplotypes. The size of the spheres is proportional to the number of individuals in each circle (numbers are shown inside the spheres). Median vectors (red open rings) represent missing or extinct species. Numbers between haplotypes indicate mutational steps. Large number of mutations were observed between *Paracoccidioides lutzii* (Plut-green) and *P. loboi* (Plob-yellow) (n = 54), between these 2 haplotypes and *P. americana* (Pame-brown) and *P. cetii* (Pcet-beige) (n = 52). Thirty-four mutations separate *P. americana* from *P. cetii*. In contrast, few mutations were observed between these four haplotypes and the remaining *Paracoccidioides* species (Pbra = *P. brasiliensis* [blue], Pres = *P. restrepiensis* [pink], Pven = *P. venzuelensis* [green]) (n = 11). Three Japanese dolphin haplotypes (Pcet = *P. cetii* [beige 1 to 3]) showed three missing or extinct haplotypes between them and the four USA dolphins (Pcet = *P. cetii* 4–7-beige) in this study.

Neighbor-joining haplotype network using 77 *Gp43* partial coding DNA sequence (Table S1) (including seven DNA sequences from dolphins in Japan = 3 and USA = 4 [accession numbers in Table S1]), showed *P. americana* (brown), *P. cetii* (beige), *P. loboi* (yellow) and *P. lutzii* (green) and the remaining *Paracoccidioides* species in separated clusters (Fig. 3). Haplotype analysis showed a relationship between *P. cetii* (beige) and *P. americana* (brown). Thirty-four mutations separated *P. cetii* and *P. americana* (Fig. 3). Two DNA sequences recovered from Japanese dolphins (Pcet3 and Pcet2-beige LC537903 and LC057206, only 110 bp of the 266 bp available at NCBI was used) displayed numerous mutations between them and the four USA dolphin DNA sequences. The third DNA sequence recovered from another dolphin in the coastal areas of Japan (Pcet1) was placed linked to the four USA dolphins (Pcew4-7), with 3 missing or extinct haplotypes and eight mutations. In this analysis, *P. loboi* (yellow) was linked to *P. lutzii* (green), with numerous mutations separating these two haplotypes (n = 54) and several missing or extinct haplotypes (red empty circles) (Fig. 3). Fifty-two mutations separated *P. americana* from *P. lutzii* and only 9 mutations were found between them and the remaining *Paracoccidioides* species (*P. brasiliensis* [blue], *P. restrepiensis* [pink] and *P. venezuelensis* [green]) (Fig. 3).

### Population STRUCTURE analysis

**Figure 4:**
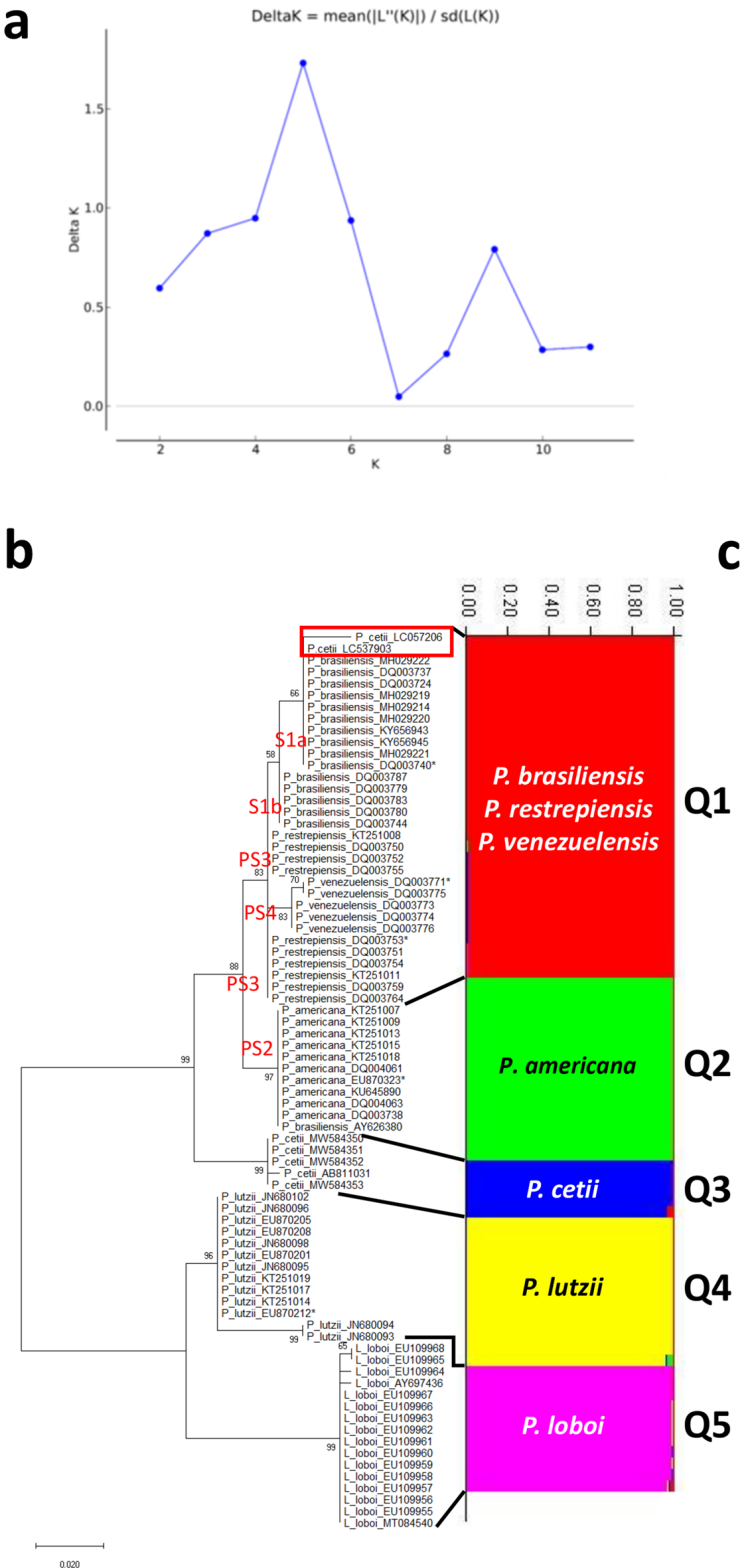
Estimation of population using LnP(D) derived ΔK for K, from 1 to 10 (K = 5), using *Gp43* DNA sequences (Panel **a**). Evolutionary analyses inferred by Maximum Likelihood of the *Gp43* DNA sequences displays five monophyletic clusters (**b**) corresponding also to five STRUCTURE populations (Q1 to Q5) (**c**). STRUCTURE clustered *P. brasiliensis* (1Sa, 1Sb), *P. restrepiensis* (PS3), *P. venezuelensis* (PS4), in population Q1 (red), whereas the remaining species (*P. americana* [PS2, green], *P. cetii* [blue], *P. lutzii* [yellow] and *P. loboi* [pink]) grouped as independent populations (Q2 to Q5). The numbers on the y-axis show the subgroup membership and the x-axis the different accessions (Q) (Panel **c**). The distribution of accessions into different populations is indicate by color and shape.

STRUCTURE software was used to determine the population structure of the *Paracoccidioides* species using *Gp43* partial coding DNA sequences (see above) (Table S1). In this analysis, the LnP(D) as well as Evanno’s ΔK showed 5 as the best K value and then, K = 5 used to build the data (Fig. 4a). The Fst average value was 0.8879 indicating high population structure. Bayesian clustering implemented in STRUCTURE using the *Gp43* (Fig. 4c), revealed subdivision of the *Paracoccidioides* species DNA sequences into five populations: *P. americana* (Q2) (PS2, green), *P. loboi* (Q5) (pink), *P. lutzii* (Q4) (yellow), and *P. cetii* (Q3) (blue). The remaining *Paracoccidioides* species (*P. brasiliensis* [1Sa, 1Sb], *P. restrepiensis* [PS3], and *P. venezuelensis* [PS4]) clustered together in a single population (Q1) (red). (Fig. 4c).

### Phylogenetic analysis

**Figure 5:**
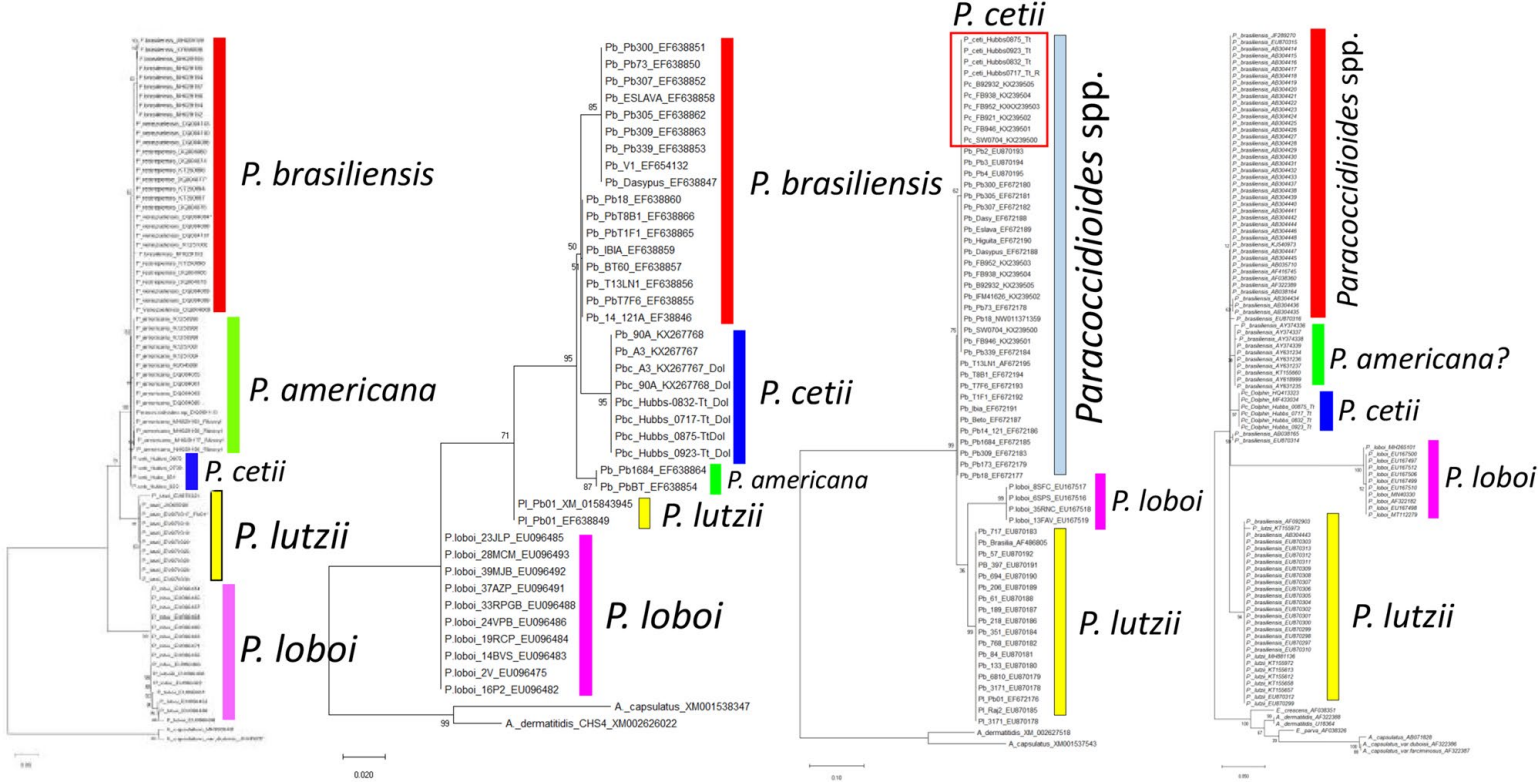
Evolutionary analyses inferred by Maximum Likelihood of the *ADP-rf*, *CHS4*, *KEX*, and ITS, DNA sequences respectively, using homologous DNA sequences from well-known dimorphic Onygenales as outgroup (Table S1). In these trees, *P. lutzii* (yellow bars) consistently grouped as monophyletic clusters sister to *P. loboi* (pink bars). Except for *Kex* DNA sequences, grouping *P. cetii* (red rectangle) with *P. brasiliensis* (sensu lato) (bluish bar), the other DNA sequences in these analyses (*ADP-rf, CHS4*, and ITS) grouped *P. cetii* (blue bars) in monophyletic clusters. *Paracoccidioides americana* (green bars) clustered as a monophyletic group using *ADP-rf* and *CHS4* DNA sequences and the other *Paracoccidioides* species (red bars) grouped in a single cluster. Using ITS DNA sequences, a poorly supported cluster was tentatively labelled as *P. americana* (green bar).

Phylogenetic trees constructed with *Gp43* partial DNA sequences of four USA dolphins, three *Gp43* partial DNA sequences from dolphins in Japan (only 110 bp was used = LC537903 and LC057206), and 70 *Gp43 Paracoccidioides* homologous DNA sequences at NCBI, showed *P. americana*, *P. cetii*, *P. loboi*, and *P. lutzii* each grouping in monophyletic clusters (Fig. 4b). The remaining *Paracoccidioides* species (1Sa, 1Sb, PS2, PS3, and PS4 in red) grouped sister to the above clusters (Fig. 4b). The *Gp43* partial DNA sequences of one of the three dolphins from Japan (AB811031) clustered together with the four DNA sequences from USA dolphins. However, two other dolphin DNA sequences also from Japan (LC537903 and LC057206) were placed with the *Gp43* DNA sequences of *P. brasiliensis* 1Sa (Fig. 4b, red rectangle). Comparative analysis of the phylogenetic results and that obtained in STRUCTURE, showed a support for the presence of five populations in both statistical approaches (Figs. 4bc) (see above). In addition, the phylogenetic evolutionary history using *ADP-rf*, *CHS4*, *KEX* partial coding homologous DNA sequences of *Paracoccidioides* species (including four USA dolphins; the Japanese dolphins do not have homologous DNA sequences in this group), and the complete ITS DNA sequences (including six dolphins from Brazil = 1, Cuba = 1, USA = 4) were investigated (Table S1). Phylogenetic analysis using the above partial coding and ITS homologous DNA sequences from dimorphic Onygenales as outgroup showed comparable threes (Fig. 5). *ADP ribosylation factor* and *CHS4* partial DNA sequences showed similar clusters to that reported with the *Gp43* DNA sequences (Figs. 4, 5). In these trees, *P. americana*, *P. cetii*, *P. loboi* and *P. lutzii* were placed in monophyletic clusters (Fig. 5). Except for *P. loboi* and *P. lutzii* forming two monophyletic clusters, *KEX* DNA sequences could not discriminate *P. cetii* from the other *Paracoccidioides* species (Fig. 5, red rectangle). The evolutionary history of the ITS DNA sequences in this study placed *P. cetii* (including dolphins in Brazil, Cuba, and the USA), *P. loboi*, and *P. lutzii* in three monophyletic clusters, but could not discriminate other *Paracoccidioides* species (Fig. 5). A poorly supported monophyletic cluster closely related to *P. cetii* was tentatively labeled as *P. americana* (Fig. 5, ITS).

### Geographic distribution, haplotype network, principal component analysis, and population structure of concatenate *Gp43* and *ADP-rf* DNA sequences

**Figure 6:**
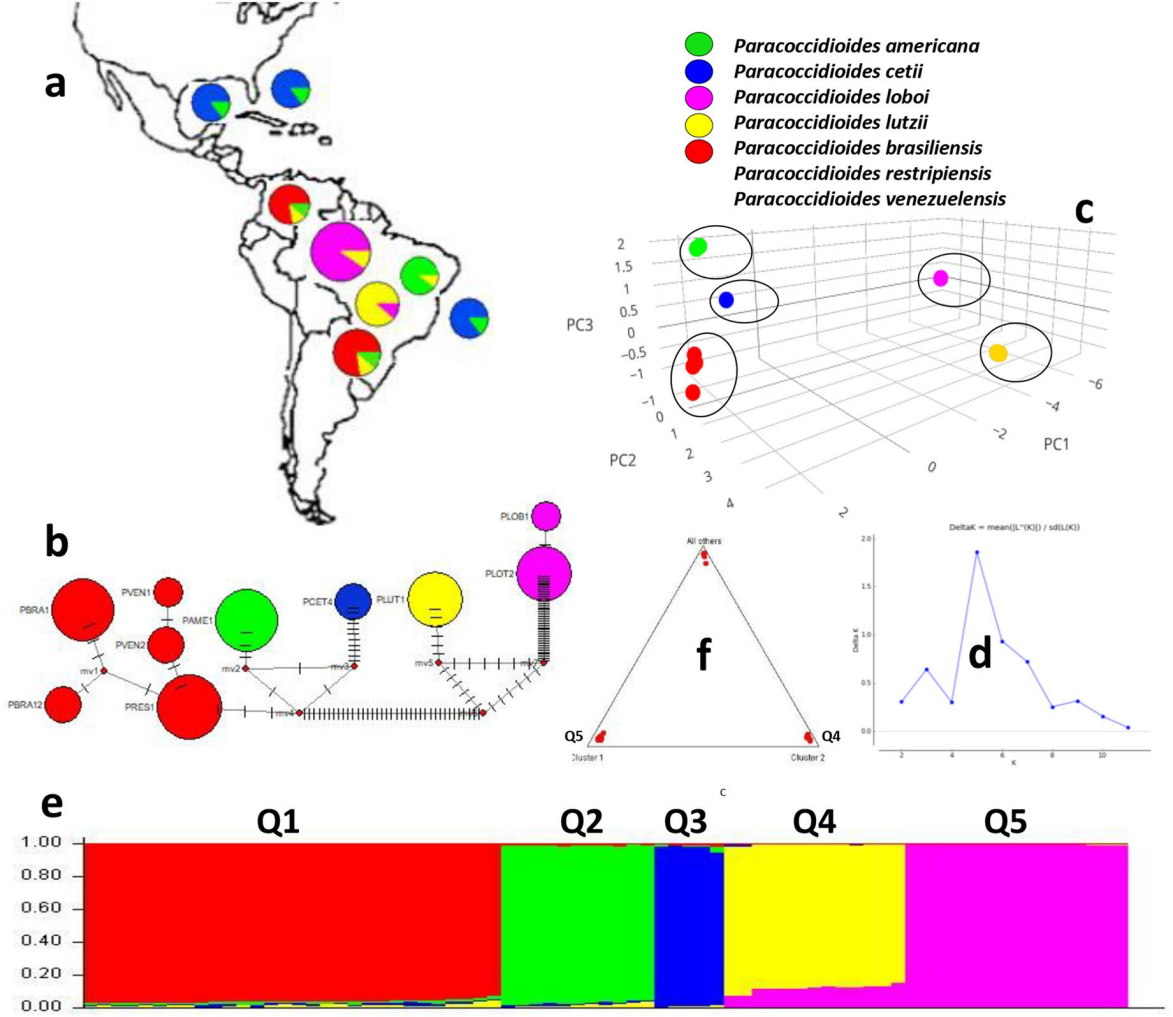
The concatenate *Gp43* and *ADP-rf* data analyses was used to perform haplotypes analysis, to locate their geographical distributions (**a**,**b**), to estimate Principal Component Analysis (PCA) (**c**) and to investigate STRUCTURE population distribution (**d**–**f**) of several *Paracoccidoides* species including *P. cetii* from dolphins and *P. loboi* from humans. The geographical distribution of the five haplotypes (**b**) is shown in Panel (**a**). Five clusters are observed in haplotype analysis (**b**) each corresponding to *P. loboi* (PLOT-pink), *P. lutzii* (PLUT yellow), *P. cetii* (PCET-blue), *P. americana* (PAME-green). The other *Paracoccidoides* species formed single haplotypes (red). Small bars between haplotypes represent mutations and the median vectors (red dots) are missing or extinct haplotypes. The size of the spheres is proportional to the number of individuals in each circle. Five-populations were also found using PCA (**c**) and STRUCTURE (**d**–**f**) analyses. Panel d shows K = 5 value used to build the concatenated data. STRUCTURE analysis (**e**) showed *P. americana* (Q2-green), *P. cetii* (Q3-blue), *P. loboi* (Pink-Q5) *P. lutzii* (yellow-Q4), and the remaining *Paracoccidioides* species (Q1-red) clustering in independent barplots (**e**). The numbers on the y-axis show the subgroup membership and the x-axis the different accessions (Q). The distribution of accessions into different populations is indicate by color and shape. The triangle plot (**f**) is an analogous result obtained from STRUCTURE software outputs. The two cluster on the base of triangle corresponds to *P. loboi* (Q5) and *P. lutzii* (Q4) DNA sequences; the top harbor the remaining species in this study.

Concatenate data of the *Gp43* plus *ADP-rf* partial coding DNA sequences (59 DNA sequences) (Table IS) using different statistical programs, consistently grouped *Paracoccidioides* species in this study into five clusters (Fig. 6). Based on recognized areas were cases of human and dolphin disease occur, the geographical distribution of these species is shown in Fig. 6a. The haplotype of *P. cetii* from dolphins is found around the coastal areas of the Americas (blue + green haplotype) (Japanese cases are not shown), whereas the haplotypes of the other *Paracoccidioides* species (*P. americana* = yellow + pink; *P. brasiliensis* = red + green + yellow; *P. lutzii* = yellow + pink) are distributed in several South America countries (Fig. 6a). *Paracoccidioides loboi* (pink + yellow) haplotype located in the Amazon basin (Fig. 6a) and other Latin American countries (not shown). The haplotype-concatenated data showed five haplotype groups comparable to the results using *Gp43* DNA sequences. (Figs. 3, 6b). Correspondingly, PCA (Fig. 6c) and STRUCTURE (Fig. 6d–f) analyses consistently grouped *Paracoccidioides* species into five populations. The LnP(D) as well as Evanno’s ΔK of the *Gp43* plus *ADP-rf* concatenate partial coding DNA sequences was K = 5 used to build the data (Fig. 6d). The triangle plot in Fig. 6f is an analogous result obtained from STRUCTURE software outputs (Q4 = *P. lutzii*, Q5 = *P. loboi* other species, top of the tringle). Similarly, phylogenetic analysis of the concatenate data, inferred by using Maximum Likelihood method and Kimura 2-parameters model, showed analogous results to the analysis using *Gp43* DNA sequences (Figs. 4b, 1c). Japanese dolphins lack *ADP-rf* DNA sequences at the NCBI thus, they were not included in this analysis. Phylogenetic analysis of the concatenated data supports the placement *P. americana*, *P. cetii*, *P. loboi* and *P. lutzii* in four monophyletic clusters (Fig. 1c).

#### Taxonomy

Based on phylogenetic and population genetics analyses of the two uncultivated pathogens of human and dolphin DNA sequences, it is now possible to place them with recognized *Paracoccidioides* species and thus, the dolphin pathogen is described as a new combination species *P. cetii*, and the human pathogen *P. loboi* as an amended *nova*.

*Paracoccidioides cetii* R. Vilela, & L. Mendoza *comb. nov*. (Basionym: *Paracoccidioides brasiliensis* var. *ceti* (R. Vilela, J. A. St. Leger, G. D. Bossart & L. Mendoza^12^).

Index Fungorum: IF558153.

**Typification**: (holotype B92-932) Slide containing *P. cetii* yeast-like cells of Hubbs-923 dolphin tissue section stained with H&E and deposited at the Michigan State University Herbarium, East Lansing Michigan. GenBank: ITS = MW566084, *ADP-rf* = MW589465, *CHS4* = MW589461, *Gp43* = MW584353, *Kex* = MW584349.

##### Etymology

Related to cetacean (Latin *cetus* = whale) aquatic mammals.

##### Description

As per Vilela and Mendoza^12^. Briefly, uncultivated fungus causing skin granulomas in several species of dolphins. In the infected tissues *P. cetii* exhibits uniform size globose to subglobose multi-budding yeast-like cells (5–10 μm), sometimes forming short or long branching chains connected by slender bridges (2–3 μm).

##### Disease nomenclature

As per Vilela et al.^23^, the term “paracoccidioidomycosis ceti” is retained. The etiologic agent of dolphins is now a different species from that reported by Jorge Lôbo causing skin disease in humans thus, the terms lacaziosis, lobomycosis, and others are no longer appropriate for this species.

*Paracoccidioides loboi* R. Vilela & L. Mendoza *amend. nov*. (Binomial originally proposed by Fonseca & Leão^10^ and Almeida & Lacaz^11^ [Basionym *Lacazia loboi* P. R. Taborda, V. A. Taborda, & M. R. McGinnis^8^]).

Index Fungorum: IF558154.

**Lectotypes, Syntypes and Description**: As per Taborda et al.^8^.

##### Disease nomenclature

Names such as Jorge Lôbo disease, lobomycosis, and others are retained. The term lacaziosis is no longer appropriate. In addition, since the term paracoccidioidomycosis has been traditionally used to describe systemic infections caused by *Paracoccidioides* species other than *P. cetii* and *P. loboi*, the term “paracoccidioidomycosis loboi” is proposed to emphasize this species is restricted to the subcutaneous tissues.

## Discussion

After 90 years of taxonomic uncertainties, using phenotypic, phylogenetic, and population genetics analyses, the two uncultivated fungi causing skin disease in humans and dolphins, long known as *Lacazia loboi*^8^, are now placed as separate species within the genus *Paracoccidioides*. Early studies using phenotypic or phylogenetic data alone erroneously placed these two fungal pathogens in different genera and species^3–8,12,13,15–17,24,25^. This trend persisted for years^2,13,16,17,25^. For instance, recent studies using several partial DNA sequences recovered from Brazilian humans with skin disease in phylogenetic analyses concluded that the genus *Lacazia*, the accepted name at that time, was an independent taxon from *Paracoccidioides* species^16,24,25^. Their phylogenetic data was correct, but their analyses missed the inclusion of DNA from the uncultivated pathogen causing skin disease in dolphins. This was an understandable mistake, since the collection and processing specimens from infected dolphins is highly regulated and the fact that the etiology of dolphins’ disease was long believed to be the same as that in humans, as shown in Fig. 1 and Table 1. Although *P. cetii* has numerous phenotypic differences with *Paracoccidioides* species (Table 1, Fig. 1), in the pass used to group them in separated clusters^2,3,7,8^, our data showed they share several phylogenetic features in common (Figs. 4, 5 and 6). With the addition of *P. cetii* DNA sequences, the phylogenetic support of closely related *Paracoccidioides* species dramatically changed. For example, *P. loboi* clustered in a monophyletic group sister to *P. lutzii*, even with the inclusion of homologous dimorphic Onygenales DNA sequences as outgroup (Figs. 4b, 5), whereas the support of monophyletic species within the genus weakened (Figs. 4, 5 and 6). More dolphin DNA sequences from different geographical locations must be sequenced to understand *P. cetii*´s true evolutionary traits.

Several studies reported geographical cryptic speciation among *Paracoccidioides* species^14,24,26–28^. In those analyses the presence of at least five species within the genus, including *P. lutzii*, was found^14,15,24,27,29,30^. Recent genome sequencing in phylogenetic analysis tend to validate these findings^26,28,29^. Although the DNA sequences of *P. loboi* were used in some of the analyses, the human skin pathogen was always placed as an independent genus from that in *Paracoccidioides* species^16,24,25^. The placement of *P. cetii* sister to *P. americana* DNA sequences in this study, indicates the use of phenotypic or phylogenetic characteristics without the inclusion of anomalous species, can lead to inaccuracies in the taxonomic and phylogenetic classification of these type of microbes. For instance, our data, using several statistical tools, consistently showed the presence of different clusters within *Paracoccidioides* species. In our analyses, *P. americana*, *P. cetii*, *P. lutzii*, and *P. loboi* were placed in monophyletic groups sister to the remaining *Paracoccidioides* species (Figs. 2, 3, 4, 5 and 6). Therefore, the addition of *P. cetii* to the genus *Paracoccidioides* not only confirmed that the genus has indeed a high level of speciation but, indicates that the concept of species delimitation in this genus must be revisited^12,31^.

Recently, Vilela et al.^16^, using phylogenetic analysis of five different genes, showed *P. loboi* shared the same ancestor with *Paracoccidioides* species. The results in our study support their proposal. The main obstacle of this hypothesis at that time was the phenotypic features of *P. loboi* (Fig. 1). However, if *P. loboi* and *P. cetii* (both uncultivated and subcutaneous pathogens) share the same ancestor with other *Paracoccidioides* species (cultivated and causing systemic infections), the likelihood that the ancestor of *Paracoccidioides* species could growth in culture, as previously suggested, is a strong possibility^16^. If this concept is correct, when in the evolutionary history of *P. cetii* and *P. loboi* they lost the capacity to grow in culture? What evolutionary pressure triggered such a change? Sadly, as is common in neglected pathogens such as *P. cetii* and *P. loboi* key questions such as these, remain without an answer. Interestingly, the uncultivated feature found in these two neglected fungi was also reported in a strain of *Histoplasma capsulatum* infecting monkeys, suggesting that an uncultivated ancestral trait in the Onygenales dimorphic fungi may be at work^32^. However, the evolutionary pressures that triggered such ancestral feature remains an enigma.

The report of new human cases of paracoccidioidomycosis loboi acquired by traveling to endemic areas^2–5,33–36^, suggests *P. loboi* may has a similar phenotype (hyphae with conidia) to the one displayed by *Paracoccidioides* species in nature and in culture. Thus, it may be present in specific ecological niches in the endemic areas (around the Amazon basin and other Latin American big rivers)^2,14,15,25^. Therefore, it is possible *P. cetii* and *P. loboi* may have a phenotype in nature similar to that of *Paracoccidioides* species (hyphae with conidia). Under this scenario, both uncultivated pathogens display a mycelia form with conidia and the classic life cycle style of dimorphic fungi in nature^25^. As is the case in other dimorphic fungi, these propagules could then contact susceptible hosts (human, dolphins) switching from hyphae → yeast thus, causing subcutaneous infections. Perhaps due to abnormalities on the molecular mechanisms of yeast → hyphae conversion (mutations?), once the hyphae → yeast conversion occurs, it cannot longer switch back from yeast to hyphal phase. However, the yeast phase of both pathogens can infect other hosts, as had been demonstrated in accidental and experimental infection with yeast-like cells from infected humans and dolphins^2,37–42^. Despite attempts made by the Broad Institute (https://www.broadinstitute.org/fungal-genome-initiative/lacazia-loboi-sequencing), only fragmented genomic information is available for *P. loboi*, and the genome of *P. cetii* is yet to be sequence. We hypothesize that the genomes of both uncultivated pathogens may hide important genomic clues that could answer this and other evolutionary questions.

Several *P. cetii* DNA sequences recovered from dolphins captured in Brazil, Cuba, Japan, and the USA are currently available in the database (Table S1)^19–23^. The complete ITS DNA sequences from Brazilian and Cuban dolphins with paracoccidioidomycosis ceti, showed high percentage of identify with the DNA sequences in this study (ITS = 100%) whereas the partial *Gp43* DNA sequences from a Japanese dolphin (471 bp) had 98.62% identity with *P. cetii* DNA sequences from dolphins captured in the Americas. During *Gp43* DNA alignment of Japanese and USA dolphins, a five nucleotides gap was consistently present in the DNA sequences of USA dolphins. Moreover, two additional 266 bp *GP43* DNA sequences extracted from a Japanese dolphin (*Lagenorhynhus obliquidens*) with paracoccidioidomycosis ceti showing, 99.62% identity with *P. brasiliensis* (sensu lato). In our analyses, these two sequences (only 110 bp could be used) clustered also with *P. brasiliensis* (Fig. 4, red rectangle). However, the same DNA sequences clustered close to *P. cetii* in haplotype analysis indicating a fragile relationship (Fig. 3). If *P. cetii* DNA sequences from Japanese dolphins are accurate, the differences in the genetic makeup of these two populations of uncultivated pathogens is intriguing and deserve further analysis. Our data suggest *P. cetii* strains causing paracoccidioidomycosis ceti in Japanese and USA dolphins, likely are evolving into two different populations.

According to Teixeira et al.^24^, the estimated time for genetic divergence in *Paracoccidioides* species was calculated around 33 million years. Although, others have questioned this result^31^, Carruthers et al.^43^, cautioned that the use of linage-specific data usually demonstrate approximate divergence time regardless of the number of loci interrogated. Nonetheless, according to these reports, *Paracoccidioides* species probably diverged from their ancestor from a fraction of a million of years (*P. restrepiensis* and *P. venezuelensis*) to 10–30 million of years (*P. lutzii* and *P. brasiliensis*, sensu lato)^24,31^. Conversely, dolphins evolved into aquatic mammals ~ 50 to 30 million years ago, around late Paleocene period (Eocene, Oligocene epochs)^44^. According to fossil records, South America at this time had a large body of water crossing from the north Atlantic Ocean to what is today Bolivia, Brazil, Ecuador, Colombia, Peru and Venezuela^45^, all endemic areas of these species^3–5,24,26,29^, that lasted for millions of years. A similar situation occurred in what is today the estuary of the Amazon River. The current location of *Paracoccidioides* species (including *P. loboi*), coincide with the locations of such geological periods, and then it is quite possible that during the time following these geological events, an ancestor of *P. cetii* first encountered dolphins entering these areas. Since humans came to South Americas only ~ 15,000-year ago^46^, likely the ancestor of *Paracoccidioides* species infected dolphin first and later humans. Whether this event had a role on the pathogenic capabilities of the genus to infect mammals is difficult to determine, nonetheless it is an intriguing possibility.

Working with uncultivated pathogens infecting the skin of mammals is challenging. Not only because collecting specimens from these species (dolphins are protected species and human cases are located in poor remote rural areas) is extremely difficult, but because open lesions usually harbor numerous environmental contaminants, which in the past had led to erroneous conclusions on the classifications of these two anomalous pathogens^2,8,15,16,25,47^. Furthermore, these unusual fungi are not in the list of neglected pathogens, thus discouraging investigators to submit proposals to funding organizations. Previous studies using *P. loboi* in phenotypic or phylogenetic analyses placed this anomalous pathogen away from the genus *Paracoccidioides*^2,4,15,16,25^. This study found that the use of phenotypic or phylogenetic approaches without the inclusion of DNA from infected dolphins, likely led previous studies to flawed data^15,16,25^. Thus, the failure of including organisms sharing a common ancestor, based in phenotypic or phylogenetic traits alone, could result in incomplete or incorrect assessment of the investigated populations. This study showed that the interpretation of taxonomic and/or phylogenetic data could be affected by missing neighboring anomalous taxa.

## Methods

### Ethics statement

Free-ranging dolphin specimens were collected under National Marine Fisheries Service Scientific Research permit number 998-1678 to Dr. Gregory D. Bossart as part of the Bottlenose Dolphin Health and Risk Assessment Project conducted in the Indian River Lagoon, Florida, and the estuarine waters of Charleston, South Carolina. Biopsied dolphin with paracoccidioidomycosis ceti infection were a gift to Biomedical Laboratory Diagnostics, Michigan State University (MSU) from previous studies^23^. The methodologies and experiments in this study were conducted following the guidelines and regulations and is ethically approved by Michigan State University, Office of Regulatory Affairs. In addition, the study was carried out in compliance with the published ARRIVE guidelines^48^.

### Phenotypic traits

Key phenotypic traits of *Paracoccidioides* species (epidemiology, distribution, etiology, laboratory finding such as histopathology, culture, experimental infections and others), were collected from data available on paracoccidioidomycosis published literature^49,50^. Likewise, the above phenotypic traits of the pathogen of dolphins were also collected from publications on the subject for the past 50 years^2,6,7,12,18–23,35,39^. The data was then used to build Table 1 displaying the most relevant phenotypic features of *Paracoccidioides* species in this study. Clinical and laboratorial data available in our facilities were also used to visualize the phenotypic traits displayed on Fig. 1.

### DNA isolation, sequencing, and genotyping

The biopsied tissues were originally sent frozen to our laboratory and manipulated according to approved guidelines^48^. Sections of the biopsied tissue containing numerous yeast-like cells by wet mount and/or histopathology were selected for DNA extraction. The dolphin skin fragments were cut into small 2 to 4 mm in diameter cubes and then ground under liquid nitrogen. The DNA from the grounded samples was treated with sodium dodecyl sulfate and digested with RNase A and protein K (Quiagen, Germantown, MD, USA) at 60 °C for 1 h. The DNA from the resulting mix was extracted with phenol/chloroform, dissolved in sterile distilled water and storage at − 80 °C, Double-stranded copies were amplified with AmpliTaq-Gold polymerase (Applied Biosystems, Branchburg, New Jersey, USA) in 25 μl volume reactions. The primers used were as per Vilela et al.^16,17,23^. Briefly, *L. loboi endoproteinase Lys/Arg-Arg* (*kex*) Llkex-1 5’TGCTTCYGGTTTGGGGTTG3' and Llkex-2 5'CACTGGARCCGTCAGCTA3' (120 bp amplicon); *L. loboi chitin synthase 4* (*CHS4*) LlCHS4-1 5'CACCACCTGTCTAAAGCT3' and LlCHS4-2 5'CGATTTCAATGTCAGAATA3' (412 bp amplicon); *L loboi ADP-ribosylation factor* (*ADP-rf*) LlRibosyl-1 5'GYCTCGATGCTGCCGGAA3' and LlRinosyl-2 5'ACGACACGGTCACGATCG3' (350 bp amplicon); *L. loboi Gp43* protein (*Gp43*) NL2 5'AACGGCTTCGACAACAGC3' and NL4 5’TAGATACATGGCGCAGTC3’ (438 bp amplicon); and the ITS primers (655 bp amplicon) of Gargas and DePriest^51^. Initially, the samples were heated at 95 °C for 10 min and then subjected to 40 cycles consisting of 1 min at 95 °C, 2 min at 60 °C, and 3 min at 72 °C, with a final extension at 72 °C for 10 min. The amplicons were purified and then sequenced in both directions with the same primers using BigDye terminator chemistry in an ABI Prism 310 genetic analyzer (Perkin-Elmer Foster City, Cal.).

### Haplotype and principal component analyses

Based on the results of previous *Paracoccidioides* species genetic and serological analyses, the *Gp43* and *ADP-rf* DNA coding regions were found to be useful in population and phylogenetic studies^12,14–16,30,52^. Thus, we selected their DNA sequence in these analyses. The amplicons alignment, mapping and SNP recognition were done in MEGA X^53^. Principal component analysis (PCA) was conducted using TASSEL v.5 software with minimum allele frequency 0.01 and additive genotype model^54^. The data were visualized with R v.4.0.3^55^. The analyzed data included 438 bp *Gp43* and the concatenate *Gp43* plus *ADP-ribosylation factor* partial DNA sequence from several *Paracoccidioides* species and the dolphin DNA sequences in this study (Table S1). In addition, diversity of haplotypes within the genus *Paracoccidioides* was also investigated using *Gp43* DNA sequences and the concatenate *Gp43* plus *ADP-rf* partial DNA sequences. The data was estimated using the software DnaSP, v7^56^ and the median-joining network was visualized in Network, v5 software (Fluxus Technology, Clare, Suffolk, England). The geographical localization of the five haplotypes in this study was based on published data where human and dolphin cases of the disease frequently occur^2–7,14,20,26,27,29,39^.

### Population STRUCTURE analysis

Spatial genetic structure was further analyzed using STRUCTURE 5.2.1^56^ which uses Bayesian algorithm to estimate probability of membership^57^. The population structure of each of the *Paracoccidioides* species in this study, based on single nucleotide polymorphism (SNPs) loci, was investigated using *Gp43* partial DNA sequences from 77 individuals and the concatenate data from *Gp43* plus *ADP-ribosylation factor* partial DNA sequences from 59 individuals (Table S1). To identify the number of populations (K) that comprises the structure of the data, the burn-in phase was set at 10,000 with the Markov Chain Monte Carlo iterations and the run duration at 50,000 using the admixture model correlating allele frequencies independently for each run. Ten runs were carried out for each value of K, with ranges from 1 to 10. For each K value, the statistical value delta K was calculated using Evanno et al.^58^ computations. The optimal K of the analysis was collected using the STRUCTURE Harvester (http://taylor0.biology.ucla.edu/structureHarvester/). Based on the LnP(D) and Evanno’s ΔK identified 5 as the best K value on both the *Gp43* partial DNA sequences and concatenate data from *Gp43* DNA plus *ADP-ribosylation facto*r partial DNA sequences. Each *Paracoccidioides* species genotype was assigned to a cluster (Q) determined by the probability of the software that a particular genotype is belong to the cluster. The cut-off probability for cluster assignment was 0.5 for more than two clusters. According to the optimum K a bar plot (sort by Q) was obtained to display the population structure among the *Paracoccidioides* spp.

### Phylogenetic analysis

Genetic diversity was also investigated, and phylogenetic trees constructed using dolphin amplified DNA sequences in this study and that available in the database including those from Japanese dolphins and from dimorphic pathogenic Onygenales, used as outgroups (Table S1). The following partial DNA sequences, *ADP-rf*, *CSH4*, *Gp43*, *kex*, and ITS, were amplified from four USA dolphins and then aligned, using MUSCLE software in MEGA X^53^, with homologous DNA sequences available at the National Center for Biotechnology Information (NCBI) (Table S1). Phylogenetic trees were constructed using MEGA X^53^. Evolutionary analyses were inferred by Maximum Likelihood and Kimura 2-parameter model^59^. The topologies generated for MP analysis were fully compatible and branches were considered supported when bootstrap values exceeded 70%. Initial tree(s) for the heuristic search were obtained automatically by applying Neighbor-Join and BioNJ algorithms to a matrix of pairwise distances estimated using the Maximum Composite Likelihood (MCL) approach, and then selecting the topology with superior log likelihood value. Codon positions included were 1st + 2nd + 3rd + Noncoding. The *Gp43* partial DNA sequence was recently described as a good marker to separate *Paracoccidioides* species^14,15,30^. Thus, we used 77 *Gp43* DNA sequences (including three dolphins. Only 110 bp was used from LC537903 and LC057206 dolphins) and 59 (*ADP-rf* + *Gp43* concatenated data) DNA sequences (dolphin *ADP-rf* DNA sequences from Japan were not available, thus they were not included in concatenated analysis) to construct the phylogenetic trees. The *ADP-rf*, *CSH4*, *Kex*, and ITS amplified dolphin DNA sequences in this study (Table S1), plus available homologous DNA sequences at the NCBI were also used to construct phylogenetic evolutionary trees using the above parameters.

## Supplementary Information

Supplementary Information.

## Data Availability

Morphological and molecular data analyzed, other than *P. cetii* in this study, had been previously published^8,9,12–17,23,24,52^. The DNA sequences and final assembly data in the manuscript have been deposited in the NCBI BioProject database under accession code PRJNA714057. Original data in this manuscript is also available by the authors on request.
