# Supplementary material for: The taxonomy of two uncultivated fungal mammalian pathogens is revealed through phylogeny and population genetic analyses
Source: Sci Rep. 2021 Sep 13;11:18119. doi: 10.1038/s41598-021-97429-7 (PMC8438014; doi:10.1038/s41598-021-97429-7)
Supplement: Supplementary file 1 — Supplementary Information. [file 41598_2021_97429_MOESM1_ESM.pdf]

Table S1 *Paracoccidioides* and homologous species in this study and their accession numbers					
Species in this study	*ADP-rf*	Gp43	*CHS4*	*KEX*	ITS
Paracoccidioides americana	KT250993	KT251007	X	X	X
Paracoccidioides americana	KT250995	KT251009	X	X	X
Paracoccidioides americana	KT250999	KT251013	X	X	X
Paracoccidioides americana	KT251001	KT251015	X	X	X
Paracoccidioides americana	KT251004	KT251018	X	X	X
Paracoccidioides americana	KU645891	KU645890	X	X	X
Paracoccidioides americana	DQ004061	DQ003736	X	X	X
Paracoccidioides americana	DQ004063	DQ003738	X	X	X
Paracoccidioides americana	EU870323	EU870210	X	X	X
Paracoccidioides americana	DQ004095	X	X	X	X
Paracoccidioides americana	DQ004113	X	X	X	X
Paracoccidioides americana	MH029193	X	X	X	X
Paracoccidioides americana	MH029186	X	X	X	X
Paracoccidioides americana	MH029177	X	X	X	X
Paracoccidioides americana	MH029196	X	X	X	X
Paracoccidioides brasiliensis	DQ004065	DQ003740	X	X	X
Paracoccidioides brasiliensis	MH029175	MH029214	X	X	X
Paracoccidioides brasiliensis	MH029180	MH029219	X	X	X
Paracoccidioides brasiliensis	MH029181	MH029220	X	X	X
Paracoccidioides brasiliensis	MH029182	MH029221	X	X	X
Paracoccidioides brasiliensis	MH029183	MH029222	X	X	X
Paracoccidioides brasiliensis	DQ004103	DQ003780	X	X	X
Paracoccidioides brasiliensis	DQ004107	DQ003783	X	X	X
Paracoccidioides brasiliensis	DQ004110	DQ003787	X	X	X
Paracoccidioides brasiliensis	DQ004069	DQ003744	X	X	X
Paracoccidioides brasiliensis	DQ004062	DQ003737	X	X	X
Paracoccidioides brasiliensis	DQ004049	DQ003724	X	X	X
Paracoccidioides brasiliensis	KY656938	KY656943	X	X	X
Paracoccidioides brasiliensis	KY656940	KY656945	X	X	X
Paracoccidioides brasiliensis	KY656936	X	X	X	X
Paracoccidioides brasiliensis	MH029159	X	X	X	X
Paracoccidioides brasiliensis	MH029162	X	X	X	X
Paracoccidioides brasiliensis	MH029163	X	X	X	X
Paracoccidioides brasiliensis	MH029164	X	X	X	X
Paracoccidioides brasiliensis	MH029165	X	X	X	X
Paracoccidioides brasiliensis	MH029166	X	X	X	X
Paracoccidioides brasiliensis	MH029184	X	X	X	X
Paracoccidioides brasiliensis	MH029185	X	X	X	X
Paracoccidioides brasiliensis	MH029197	X	X	X	X
Paracoccidioides brasiliensis	X	AY626380	X	X	X
Paracoccidioides brasiliensis	X	DQ003772	X	X	X
Paracoccidioides brasiliensis	X	X	EF638832	X	X
Paracoccidioides brasiliensis	X	X	EF638846	X	X
Paracoccidioides brasiliensis	X	X	EF638855	X	X
Paracoccidioides brasiliensis	X	X	EF638857	X	X
Paracoccidioides brasiliensis	X	X	EF638864	EF672185	X
Paracoccidioides brasiliensis	X	X	EF638859	EF672191	X
Paracoccidioides brasiliensis	X	X	EF638865	EF672192	X
Paracoccidioides brasiliensis	X	X	EF638866	EF672194	X
Paracoccidioides brasiliensis	X	X	EF638860	EF672177	AF322389
Paracoccidioides brasiliensis	X	X	EF638847	EF672188	AY631234

Paracoccidioides brasiliensis	X	X	EF638856	EF672195	X
Paracoccidioides brasiliensis	X	X	EF638863	EF672183	X
Paracoccidioides brasiliensis	X	X	EF638862	EF672181	X
Paracoccidioides brasiliensis	X	X	EF638858	EF672189	X
Paracoccidioides brasiliensis	X	X	EF638852	EF672182	X
Paracoccidioides brasiliensis	X	X	EF638850	EF672178	X
Paracoccidioides brasiliensis	X	X	EF638851	EF672180	X
Paracoccidioides brasiliensis	X	X	X	AF672193	EU870315
Paracoccidioides brasiliensis	X	X	X	EF672179	X
Paracoccidioides brasiliensis	X	X	X	EF672183	X
Paracoccidioides brasiliensis	X	X	X	EF672184	X
Paracoccidioides brasiliensis	X	X	X	EF672186	X
Paracoccidioides brasiliensis	X	X	X	EF672187	X
Paracoccidioides brasiliensis	X	X	X	EF672190	X
Paracoccidioides brasiliensis	X	X	X	EF672194	X
Paracoccidioides brasiliensis	X	X	X	EU870193	X
Paracoccidioides brasiliensis	X	X	X	EU870194	X
Paracoccidioides brasiliensis	X	X	X	EU870195	EU870316
Paracoccidioides brasiliensis	X	X	X	KX239500	X
Paracoccidioides brasiliensis	X	X	X	KX239501	X
Paracoccidioides brasiliensis	X	X	X	KX239502	AB304420
Paracoccidioides brasiliensis	X	X	X	KX239503	X
Paracoccidioides brasiliensis	X	X	X	KX239504	X
Paracoccidioides brasiliensis	X	X	X	KX239505	X
Paracoccidioides brasiliensis	X	X	X	X	AB035710
Paracoccidioides brasiliensis	X	X	X	X	AB038164
Paracoccidioides brasiliensis	X	X	X	X	AB304414
Paracoccidioides brasiliensis	X	X	X	X	AB304415
Paracoccidioides brasiliensis	X	X	X	X	AB304416
Paracoccidioides brasiliensis	X	X	X	X	AB304417
Paracoccidioides brasiliensis	X	X	X	X	AB304418
Paracoccidioides brasiliensis	X	X	X	X	AB304419
Paracoccidioides brasiliensis	X	X	X	X	AB304421
Paracoccidioides brasiliensis	X	X	X	X	AB304422
Paracoccidioides brasiliensis	X	X	X	X	AB304423
Paracoccidioides brasiliensis	X	X	X	X	AB304424
Paracoccidioides brasiliensis	X	X	X	X	AB304425
Paracoccidioides brasiliensis	X	X	X	X	AB304426
Paracoccidioides brasiliensis	X	X	X	X	AB304427
Paracoccidioides brasiliensis	X	X	X	X	AB304428
Paracoccidioides brasiliensis	X	X	X	X	AB304429
Paracoccidioides brasiliensis	X	X	X	X	AB304430
Paracoccidioides brasiliensis	X	X	X	X	AB304431
Paracoccidioides brasiliensis	X	X	X	X	AB304432
Paracoccidioides brasiliensis	X	X	X	X	AB304433
Paracoccidioides brasiliensis	X	X	X	X	AB304434
Paracoccidioides brasiliensis	X	X	X	X	AB304435
Paracoccidioides brasiliensis	X	X	X	X	AB304436
Paracoccidioides brasiliensis	X	X	X	X	AB304437
Paracoccidioides brasiliensis	X	X	X	X	AB304438
Paracoccidioides brasiliensis	X	X	X	X	AB304439
Paracoccidioides brasiliensis	X	X	X	X	AB304440
Paracoccidioides brasiliensis	X	X	X	X	AB304441

Paracoccidioides brasiliensis	X	X	X	X	AB304442
Paracoccidioides brasiliensis	X	X	X	X	AB304444
Paracoccidioides brasiliensis	X	X	X	X	AB304445
Paracoccidioides brasiliensis	X	X	X	X	AB304446
Paracoccidioides brasiliensis	X	X	X	X	AB304447
Paracoccidioides brasiliensis	X	X	X	X	AB304448
Paracoccidioides brasiliensis	X	X	X	X	AF038360
Paracoccidioides brasiliensis	X	X	X	X	AF416745
Paracoccidioides brasiliensis	X	X	X	X	AY374336
Paracoccidioides brasiliensis	X	X	X	X	AY374337
Paracoccidioides brasiliensis	X	X	X	X	AY374339
Paracoccidioides brasiliensis	X	X	X	X	AY618999
Paracoccidioides brasiliensis	X	X	X	X	AY631235
Paracoccidioides brasiliensis	X	X	X	X	AY631236
Paracoccidioides brasiliensis	X	X	X	X	AY631237
Paracoccidioides brasiliensis	X	X	X	X	JF289270
Paracoccidioides brasiliensis	X	X	X	X	KJ540973
Paracoccidioides brasiliensis	X	X	X	X	KT155660
Paracoccidioides venezuelensis	DQ004094	DQ003771	X	X	X
Paracoccidioides venezuelensis	DQ004096	DQ003773	X	X	X
Paracoccidioides venezuelensis	DQ004098	DQ003774	X	X	X
Paracoccidioides venezuelensis	DQ004099	DQ003775	X	X	X
Paracoccidioides venezuelensis	DQ004100	DQ003776	X	X	X
Paracoccidioides venezuelensis	KT251002	X	X	X	X
Paracoccidioides venezuelensis	DQ004069	X	X	X	X
Paracoccidioides venezuelensis	DQ004103	X	X	X	X
Paracoccidioides venezuelensis	DQ004107	X	X	X	X
Paracoccidioides restripiensis	DQ004074	DQ003750	X	X	X
Paracoccidioides restripiensis	DQ004075	DQ003751	X	X	X
Paracoccidioides restripiensis	DQ004076	DQ003752	X	X	X
Paracoccidioides restripiensis	DQ004077	DQ003753	X	X	X
Paracoccidioides restripiensis	DQ004078	DQ003754	X	X	X
Paracoccidioides restripiensis	DQ004079	DQ003755	X	X	X
Paracoccidioides restripiensis	DQ004088	DQ003764	X	X	X
Paracoccidioides restripiensis	DQ004089	DQ003765	X	X	X
Paracoccidioides restripiensis	DQ004090	DQ003766	X	X	X
Paracoccidioides restripiensis	DQ004080	X	X	X	X
Paracoccidioides restripiensis	DQ004094	X	X	X	X
Paracoccidioides restripiensis	DQ004066	X	X	X	X
Paracoccidioides restripiensis	KT250994	X	X	X	X
Paracoccidioides restripiensis	KT250996	X	X	X	X
Paracoccidioides restripiensis	KT250997	X	X	X	X
Paracoccidioides restripiensis	KT250998	X	X	X	X
Paracoccidioides lutzii	X	JN680093	X	X	X
Paracoccidioides lutzii	X	JN680096	X	X	X
Paracoccidioides lutzii	X	JN680102	X	X	X
Paracoccidioides lutzii	X	KT251014	X	X	X
Paracoccidioides lutzii	X	KT251017	X	X	X
Paracoccidioides lutzii	X	KT251019	X	X	X
Paracoccidioides lutzii	EU870317	EU870212	EF638849	EF672176	AF092903
Paracoccidioides lutzii	EU870319	EU870208	X	X	X
Paracoccidioides lutzii	EU870321	EU870201	X	X	X
Paracoccidioides lutzii	EU870333	EU870205	X	X	X

Paracoccidioides lutzii	JN680080	JN680094	X	X	X
Paracoccidioides lutzii	JN680081	JN680095	X	X	X
Paracoccidioides lutzii	JN680087	JN680098	X	X	X
Paracoccidioides lutzii	EU870318	X	X	EU870179	EU870299
Paracoccidioides lutzii	EU870320	X	X	EU870183	EU870311
Paracoccidioides lutzii	EU870322	X	X	X	EU870298
Paracoccidioides lutzii	EU870326	X	X	EU870187	EU870305
Paracoccidioides lutzii	EU870328	X	X	X	EU870307
Paracoccidioides lutzii	EU870330	X	X	EU870192	EU870309
Paracoccidioides lutzii	X	X	X	AF486805	X
Paracoccidioides lutzii	X	X	X	EU870178	EU870300
Paracoccidioides lutzii	X	X	X	EU870180	EU870310
Paracoccidioides lutzii	X	X	X	EU870181	EU870313
Paracoccidioides lutzii	X	X	X	EU870182	EU870303
Paracoccidioides lutzii	X	X	X	EU870184	EU870302
Paracoccidioides lutzii	X	X	X	EU870186	EU870304
Paracoccidioides lutzii	X	X	X	EU870188	EU870306
Paracoccidioides lutzii	X	X	X	EU870189	EU870301
Paracoccidioides lutzii	X	X	X	EU870190	EU870308
Paracoccidioides lutzii	X	X	X	EU870191	EU870312
Paracoccidioides lutzii	X	X	X	X	EU870297
Paracoccidioides lutzii	X	X	X	X	MH881136
Paracoccidioides lutzii	X	X	X	X	KT155972
Paracoccidioides lutzii	X	X	X	X	KT155613
Paracoccidioides lutzii	X	X	X	X	KT155612
Paracoccidioides lutzii	X	X	X	X	KT155657
Paracoccidioides lutzii	X	X	X	X	KT155658
Paracoccidioides lutzii	X	X	X	X	KT155973
Paracoccidioides loboi	EU096473	EU109958	X	EU167517	X
Paracoccidioides loboi	EU096466	EU109959	EU096491	X	EU167499
Paracoccidioides loboi	EU096462	EU109960	X	X	AF322182
Paracoccidioides loboi	EU096457	EU109962	EU096475	X	EU167500
Paracoccidioides loboi	EU096460	EU109963	X	X	EU167512
Paracoccidioides loboi	EU096472	EU109964	EU096483	X	X
Paracoccidioides loboi	EU096459	EU109965	X	X	EU167506
Paracoccidioides loboi	EU096458	EU109966	X	EU167519	X
Paracoccidioides loboi	EU096456	EU109967	X	EU167516	X
Paracoccidioides loboi	EU096470	EU109968	EU096492	X	X
Paracoccidioides loboi	X	MT084540	X	X	X
Paracoccidioides loboi	X	EU109961	X	X	X
Paracoccidioides loboi	X	AY697436	X	X	X
Paracoccidioides loboi	X	EU109955	EU096486	X	EU167497
Paracoccidioides loboi	X	EU109956	X	EU167518	EU167510
Paracoccidioides loboi	X	EU109957	EU096484	X	X
Paracoccidioides loboi	X	X	X	X	MH265101
Paracoccidioides loboi	X	X	X	X	MN403304
Paracoccidioides loboi	X	X	X	X	MT112279
Paracoccidioides loboi	X	X	EU096493	X	EU167498
Paracoccidioides loboi	X	X	EU096488	X	X
Paracoccidioides loboi	X	X	EU096482	X	X
Paracoccidioides loboi	X	X	EU096485	X	X
Paracoccidioides loboi	EU096454	X	X	X	X
Paracoccidioides loboi	EU096455	X	X	X	X

Paracoccidioides loboi	EU096464	X	X	X	X
Paracoccidioides loboi	EU096463	X	X	X	X
Paracoccidioides loboi	EU096471	X	X	X	X
Paracoccidioides loboi	EU096461	X	X	X	X
Paracoccidioides loboi	EU096468	X	X	X	X
Paracoccidioides cetii Hubbs_875	MW589462	MW584350	MW589458	MW584346	MW566081
Paracoccidioides cetii Hubbs_938	MW589463	MW584351	MW589459	MW584347	MW566082
Paracoccidioides cetii Hubbs_738	MW589464	MW584352	MW589460	MW584348	MW566083
Paracoccidioides cetii Hubbs_923	MW589465	MW584353	MW589461	MW584349	MW566084
Paracoccidioides cetii	X	X	X	KX239504	X
Paracoccidioides cetii	X	X	X	KX239505	X
Paracoccidioides cetii	X	X	X	KX239500	X
Paracoccidioides cetii	X	X	X	KX239501	X
Paracoccidioides cetii	X	X	X	KX239502	X
Paracoccidioides cetii	X	X	X	KX239503	X
Paracoccidioides cetii	X	X	KX267767	X	X
Paracoccidioides cetii	X	X	KX267768	X	X
Paracoccidioides cetii	X	X	X	X	MF433034
Paracoccidioides cetii	X	X	X	X	HQ413323
Paracoccidioides cetii	X	AB811031	X	X	X
Paracoccidioides cetii	X	LC057206	X	X	X
Paracoccidioides cetii	X	LC537903	X	X	X
*Histoplasma capsulatum**	JK443637	X	X	X	X
*Histoplasma capsulatum**	MK893640	X	X	X	X
*Ajellomyces capsulatus**	X	X	X	XM001537543	X
*Ajellomyces dermatitidis**	X	X	X	XM002627518	X
*Ajellomyces capsulatus**	X	X	XM001538347	X	X
*Ajellomyces dermatitidis**	X	X	XM002626022	X	X
*Ajellomyces capsulatus**	X	X	X	X	AB071828
*A. capsulatus var duboisii**	X	X	X	X	AF322386
*A. capsulatus var farciminosum**	X	X	X	X	AF322387
*Aajellomyces dermatitidis**	X	X	X	X	AF322388
*Ajellomyces dermatitidis**	X	X	X	X	U18364
*Emmonsia parva**	X	X	X	X	AF038326
*Emmonsia crescens**	X	X	X	X	AF038351

X= DNA sequences not included or not available

*DNA sequences used as outgroup
